# Plasma Adsorption Perfusion (BR‐350) Versus Open Albumin Dialysis (OPAL) for Hyperbilirubinemia in Hepatic Failure in Cirrhotic Patients

**DOI:** 10.1111/aor.70090

**Published:** 2026-01-09

**Authors:** Justa Friebus‐Kardash, Alica Ochs, Amina Louzi, Andreas Kribben, Bartosz Tyczynski, Jassin Rashidi‐Alavijeh, Andreas Schütte, Hartmut H. Schmidt, Amos Zeller

**Affiliations:** ^1^ Department of Nephrology University of Duisburg‐Essen, University Hospital Essen Essen Germany; ^2^ Department of Intensive Care Medicine I University Hospital Essen Essen Germany; ^3^ Department of Gastroenterology, Hepatology and Transplant Medicine University of Duisburg‐Essen, University Hospital Essen Essen Germany

**Keywords:** adsorber BR‐350, bile acids, bilirubin, blood pressure, liver failure, open albumin dialysis, plasma adsorption perfusion

## Abstract

**Background:**

In the last decades, various extracorporeal liver support systems were developed for hepatic failure with hyperbilirubinemia with the aim to clear the blood from protein‐bound toxic molecules. Open albumin dialysis (OPAL) is a complex and expensive system that requires addition of human albumin. Plasma adsorption perfusion (PAP) using the anion exchange resin adsorber, BR‐350, is an alternative liver support method that does not need additional blood products and is more cost‐effective and less time‐consuming.

**Methods:**

At the University Hospital Essen, PAP using BR‐350 was performed in a cohort of 9 patients with a mean of 6 sessions per patient. In a retrospective analysis, we compared the detoxification ability of PAP with that of the OPAL system conducted among 24 patients and with standard medical treatment (SMT) including hemodialysis that was performed among 24 patients. In addition, the technical effectiveness of a single session of PAP with BR‐350 was compared with OPAL among 12 patients who were treated with both methods in a crossover design.

**Results:**

The first single session (30.7.3 ± 13.5 mg/dL to 25.8 ± 13.4 mg/dL, *p* = 0.02) and the complete therapy (30.7 ± 13.5 mg/dL to 16.2 ± 6.3 mg/dL, *p* = 0.004) with PAP using BR‐350 resulted in a statistically significant decrease of bilirubin concentrations. The median relative reduction of bilirubin achieved at the end of liver support treatment was comparable between PAP and OPAL (47% vs. 40%, *p* = 0.29). PAP was associated with a higher bilirubin reduction than the SMT plus dialysis (47% vs. −30%, *p* = 0.0001). The crossover comparison between the single session of PAP using BR‐350 and OPAL revealed similar mean relative reduction rates of bilirubin (11% vs. 10%, *p* = 0.81). The single session of OPAL was associated with a more pronounced decrease of alkaline phosphatase, gamma‐glutamyltransferase, hemoglobin, platelets, and leucocytes compared to PAP.

**Conclusions:**

Both studied methods had comparable efficacy in reducing bilirubin in our studied patients in contrast to the retrospective control group. Since other substrates may also be relevant in treating liver failure, more studies are required. Patients with concomitant renal failure benefit from OPAL, whereas PAP might be more eligible for patients with a high risk of bleeding.

## Graphical abstract

The therapy with PAP using BR‐350 resulted in a significant decrease of bilirubin concentrations. The median relative reduction of bilirubin was comparable between PAP and OPAL (47% vs. 40%, *p* = 0.29). The crossover comparison between the single session of PAP using BR‐350 and OPAL revealed similar relative reduction rates of bilirubin.

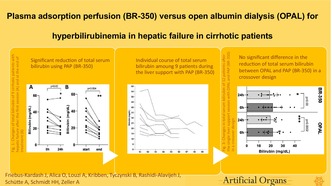

## 1 Background

Liver failure is complex and can result in multiple organ failure. Among different treatment strategies, artificial extracorporeal liver support techniques were developed in recent decades. The idea is to remove lipid‐soluble, protein‐bound toxic compounds in hepatic failure [1, 2]. Bilirubin is a surrogate marker for lipophilic toxic substances accumulating during liver failure and therapeutic interventions to lower bilirubin may improve the outcome of patients [1, 2, 3, 4]. Extracorporeal detoxification of the toxins using liver support systems allows limiting the ongoing damage of liver tissue and may create a favorable environment for the regeneration of hepatic function [1, 2, 3, 4]. Thus, artificial extracorporeal systems are used for the purpose to bridge patients with liver failure of different etiologies to liver transplant or to spontaneous recovery [1, 2, 3, 4]. The most commonly used artificial extracorporeal liver support techniques are based on plasmapheresis, albumin dialysis, and hemoadsorption [1, 2, 3, 4].

Plasmapheresis is an effective and quickly available liver support modality [1, 4]. However, it is a crude unselective liver support technique that eliminates a number of physiological plasma proteins besides bilirubin and bile acids that might be disadvantageous in case of reduced protein synthesis capacity due to hepatic dysfunction [1, 4]. In addition, plasma exchange requires substitution of large volumes of fresh frozen plasma that is associated with high costs and possible side effects such as embolic events, infections, and anaphylactic reactions [1, 4]. On the other hand, Molecular Adsorbent Recirculating System (MARS) as the best studied method of albumin dialysis comprises two different circuits, a blood circuit and an albumin circuit, and was shown in several studies to successfully remove lipophilic as well as hydrophilic substances from blood circulation, in particular bilirubin, bile acids, creatinine, urea, ammonia, lactate, aromatic amino acids and paracetamol [2, 5, 6, 7, 8, 9]. In MARS, human albumin acts as the carrier for the protein‐bound, lipophilic toxins and regenerates in a closed recycling system [2]. During albumin recycling, the toxins bound to human albumin are released and adsorbed by charcoal and anion‐exchange resin absorbers in the albumin circuit [2]. Beneficial effects on hepatic encephalopathy, hepatorenal syndrome, and hyperbilirubinemia were shown for the extracorporeal liver support therapy with MARS [10]. Gerth et al. demonstrated in the retrospective analysis a short‐term survival advantage of MARS combined with standard treatment among 47 patients with acute‐on chronic‐liver failure (ACLF) in comparison to 54 ACLF patients who received only standard treatment [11]. Otherwise, MARS therapy did not significantly improve the 28‐day survival among hemodynamically and respiratory stable 22 patients with acute liver failure (ALF) and 10 patients with graft dysfunction after liver transplant when comparing to standard medical treatment (31 ALF patients and 10 patients with graft dysfunction) [12]. The open albumin dialysis (OPAL) system is an advanced device for albumin dialysis and further development of MARS having more efficient albumin‐bound detoxification than MARS because of increased surface area of the charcoal absorber [13]. In our previous work, we observed an effective removal of bilirubin and serum creatinine and improvement of the MELD score after application of OPAL in a cohort of patients with ACLF [14]. Nevertheless, equipping of the MARS or OPAL machine is complex, time consuming and requires special expertise and well‐trained personnel causing high costs [13, 14].

The plasma adsorption perfusion (PAP) using the anion exchange resin adsorption column, BR‐350, is an alternative option for selective detoxification of bilirubin and bile acids [2, 15, 16, 17, 18, 19]. During the PAP with BR‐350, plasma is separated by a plasma‐filter in the first step and then passed through adsorber BR‐350 made of the resin styrenedivinylbenzene with trimethylbenzylammonium as the ligand [15, 16]. Recent case reports and case series reported a high affinity of the BR‐350 column to bilirubin and bile acids and revealed PAP with BR‐350 as a safe and effective liver support treatment for hepatic dysfunction due to several etiologies [2, 15, 16, 17, 18, 19]. In contrast to plasmapheresis and albumin dialysis, PAP with BR‐350 does not need additional application of foreign blood products, such as fresh frozen plasma or human albumin, and is relatively easy to perform without side effects that enhance the safety and the cost‐effectiveness of this liver support method [15, 16, 17, 18, 19].

In the current study, we first summarized our center experience with PAP using BR‐350 in a cohort of 9 patients with preexisting liver cirrhosis treated for different indications. Moreover, we aimed to compare PAP with BR‐350 with extracorporeal liver support therapy with OPAL for hepatic dysfunction due to diverse reasons as well as with standard medical treatment (SMT) in particular in terms of detoxification capacity.

## 2 Methods

### 2.1 Study Population

We conducted a retrospective analysis on 9 cirrhotic patients who were exclusively treated with PAP using BR‐350 as a liver support device between April 2016 and November 2023 2024 at our center because of hepatic failure that occurred due to various reasons (Table 1). Furthermore, we retrospectively compared the cohort of 9 patients with a cohort of 24 cirrhotic patients presenting with hepatic failure of different origin who were treated with OPAL as an alternative liver support method between December 2021 and October 2023 at our center. To gain further insights we then established retrospectively a cohort of 24 cirrhotic patients with no extracorporeal liver support as SMT including both systemic application of human albumin and vasoconstrictor terlipressin plus intermittent hemodialysis. Patients analyzed were hospitalized between July 2012 and November 2022 due to hepatorenal syndrome. Additionally, we performed a retrospective comparative analysis on another cohort consisting of a total of 12 cirrhotic patients who were treated with both liver support devices, PAP as well as OPAL, between March 2022 and May 2024 at our center. Thus, we compared the first single session of PAP and OPAL in a crossover manner. The time interval between the single OPAL session and PAP session lasted from 24 to 72 h. All study patients were hemodynamically stable throughout the entire liver support treatment and were treated on the normal ward. The retrospective monocentric study was approved by the local Ethics Board of the University Hospital Essen (23‐11405‐BO).

**TABLE 1:** Baseline clinical and laboratory values of 9 patients treated exclusively with PAP using BR‐350 between April 2016 and November 2023.

Variable	PAP with BR‐350
	*n* = 9
Women, *n* (%)	5 (56)
Age (years)	54 (46–61)
Number of session	6 (4–7)
MELD‐Na score (points)	26 (22–37)
Hepatorenal syndrome, *n* (%)	3 (33)
Hepatic encephalopathy, *n* (%)	3 (33)
Dialysis before therapy initiation, *n* (%)	0 (0)
Administration to the ICU before therapy initiation, *n* (%)	2 (22)
Etiology of liver dysfunction	
ACLF (grade 1–2), *n* (%)	6 (67)
Decompensation of liver cirrhosis, *n* (%)	3 (33)
Baseline laboratory values	
Bilirubin (mg/dL)	29.2 (19.1–42.6)
ALT (U/L)	74 (51–197)
AST (U/L)	125 (109–144)
AP (U/L)	193 (152–435)
GGT (U/L)	135 (60–325)
LDH (U/L)	256 (186–358)
INR	1.41 (1.21–1.64)
PTT (msec)	38.8 (29.6–43.0)
Albumin (mg/dL)	3.6 (3.1–4.8)
Serum creatinine (mg/dL)	1.08 (0.87–2.76)
eGFR (MDRD, mL/min/1.73 m^2^)	60 (28–60)
Urea‐N (mg/dL)	36.4 (24.3–48.5)
Hemoglobin (g/dL)	10.2 (7.3–13.0)
Platelets (/nL)	116 (69–195)
Leukocytes (/nL)	9.3 (7.1–13.8)

*Note:* Values are presented as medians with interquartile range.

Abbreviations: ACLF, acute‐on‐chronic liver failure; ALT, alanine transaminase; AP, alkaline phosphatase; AST, aspartate transaminase; eGFR, estimated glomerular filtration rate; GGT, gamma‐glutamyltransferase; ICU, intensive care unit; INR, international normalized ratio; L, liter; LDH, lactate dehydrogenase; MDRD, modification of diet in renal disease; MELD‐Na, model for end‐stage liver disease‐ sodium; PAP, plasma adsorption perfusion; PTT, partial thromboplastin time; U, unit; urea‐N, urea nitrogen.

Blood samples were collected in the context of clinical routine before the initiation of extracorporeal liver support and before and after every extracorporeal treatment session (within the first 24 h after the completion of each liver support session), and total serum bilirubin levels, transaminases, coagulation parameters, albumin, leucocytes, platelets, hemoglobin, serum creatinine, and urea nitrogen were determined. In the cohort of 12 patients who received extracorporeal therapy with PAP and OPAL at the same time period, levels of bilirubin and bile acids were directly measured before and after the treatment session. Biochemical measurements were performed using standard laboratory procedures. Clinical data of the patients were obtained by retrospective review of electronic medical records.

### 2.2 Extracorporeal Liver Support Therapies

PAP using BR‐350 is based on the concept of plasma adsorption. Patient's blood was purified and separated by a plasmafilter (Plasmaflo OP‐08, Diamed, Cologne, Germany) using a plasma exchange monitor (Plasauto SIGMA, Diamed, Cologne, Germany) and afterward perfused through an anion exchange resin column (Plasorba BR‐350, Diamed, Cologne, Germany). The effluent plasma was returned to the patient. Unfractionated heparin was used as the standard anticoagulant. The heparin dose was monitored at the bedside by checking the activated clotting time (ACT). In a single treatment session, 6–8 L of plasma were separated with a blood flow ranging between 100 and 150 mL/min and plasma passed through the BR‐350 adsorber with a plasma flow rate of 30–40 mL/min.

Indeed, OPAL was based on the concept of albumin dialysis. The OPAL device was attached to a standard hemodialysis machine, the FMC MultiFiltrate (Fresenius Medical Care AG, Bad Homburg, Germany), with a Maxicycler absorber (Albutec, Rostock, Germany). The blood was dialyzed across an albumin impermeable high‐flux dialyzer (FX, Fresenius, Germany) and perfused through a combination of charcoal and anion‐exchange resins in the secondary albumin circuit. The albumin circuit was primed with 400 mL of a 20% human albumin solution. The OPAL device was built according to the manufacturer's instructions. The average treatment time was 360 min. Blood flow rates were set at between 100 and 150 mL/min, and albumin flow rates were set at 250 mL/min. Anticoagulation was maintained by the application of regional citrate.

Catheterization of internal jugular vein or femoral vein with a dual‐lumen catheter was considered a temporary vascular access.

### 2.3 Statistical Analysis

Categorical variables were presented as numbers and percentages, and continuous variables were given as medians with interquartile ranges. The Wilcoxon test was conducted to determine differences between pre‐ and post‐therapy values. The two‐tailed *χ* ^*2*^ test for categorical variables and the Mann–Whitney test for not normally distributed quantitative data were used to evaluate differences between extracorporeal liver support treatment with PAP using BR‐350 and OPAL or SMT. All *p* values are two‐tailed, and statistical significance was assumed for *p* values ≤ 0.05. All calculations were made with GraphPad Prism version 6 (GraphPad Software Inc., La Jolla, CA, USA).

## 3 Results

### 3.1 Detoxification Ability of PAP Using BR‐350

First, we analyzed removal of bilirubin and changes of relevant laboratory values in a cohort of 9 patients with preexisting liver cirrhosis who were exclusively treated with PAP using the adsorber BR‐350 as an artificial liver support method. The baseline characteristics of the cohort are depicted in Table 1. ACLF and decompensation of preexisting liver cirrhosis were the indications for the application of PAP with BR‐350 (Table 1). The median number of PAP sessions was six, ranging from four to seven sessions in the entire cohort (Table 1). The median MELD‐Na score among 9 patients was 26 points (Table 1). The median bilirubin concentration before initiation of PAP using BR‐350 was 29.2 mg/dL (Table 1). Another liver support device was not applied before the initiation of PAP. Three (33%) of 9 patients exhibited a hepatorenal syndrome with impaired renal function (Table 1). The majority of patients within the cohort had normal renal function with a median serum creatinine of 1.08 mg/dL and estimated GFR of at least 60 mL/min/1.73 m^2^ according to the MDRD formula (Table 1).

**FIGURE 1:**
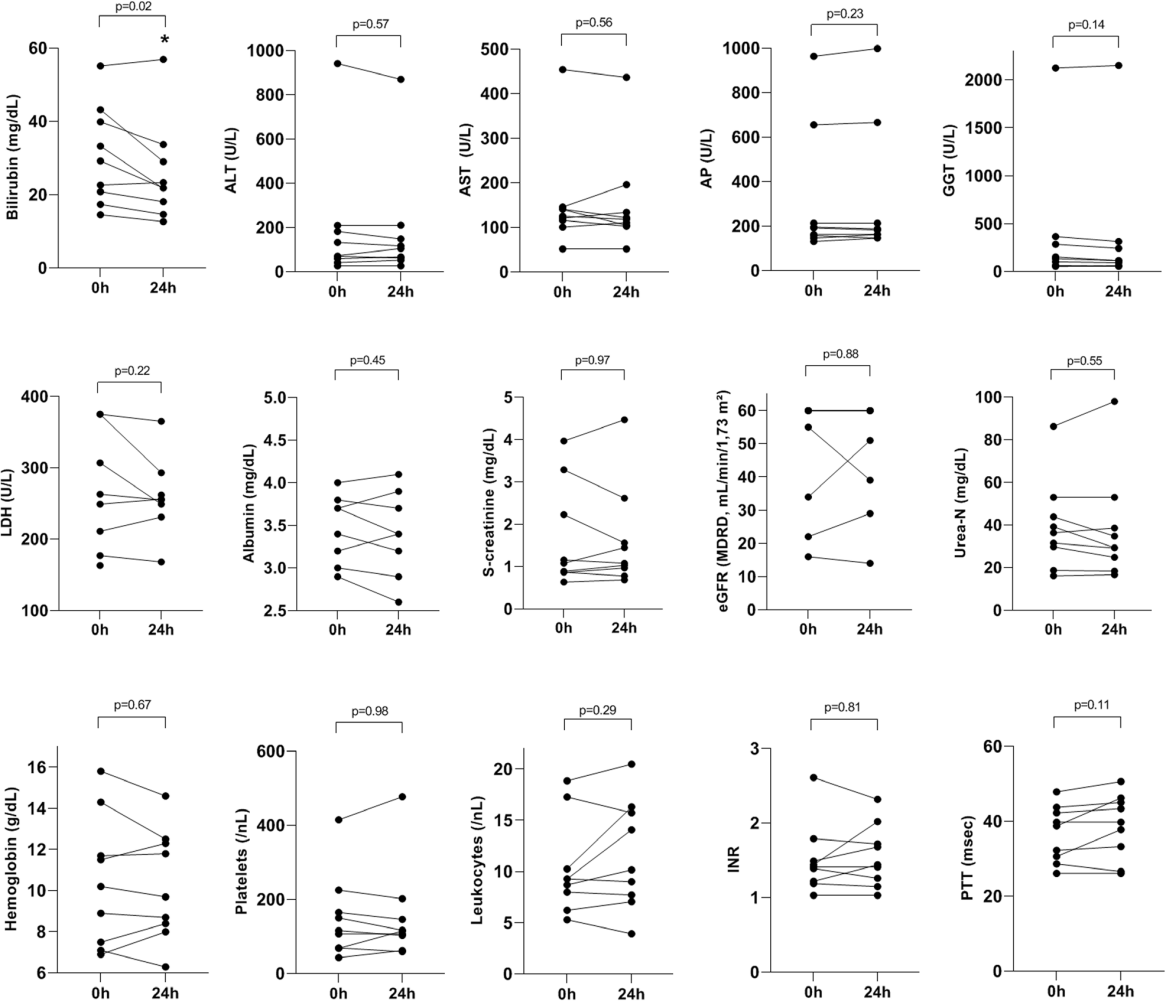
Changes in biochemical variables of 9 patients after the first session of PAP using BR‐350. ALT, alanine transaminase; AP, alkaline phosphatase; AST, aspartate transaminase; INR, international normalized ratio; eGFR, estimated glomerular filtration rate; GGT, gamma‐glutamyltransferase; L, liter; LDH, lactate dehydrogenase; MDRD, modification of diet in renal disease; MELD‐Na, model for end‐stage liver disease‐ sodium; PAP, plasma adsorption perfusion; PTT, partial thromboplastin time; s‐creatinine, serum creatinine; U, unit; urea‐N, urea nitrogen.

**FIGURE 2:**
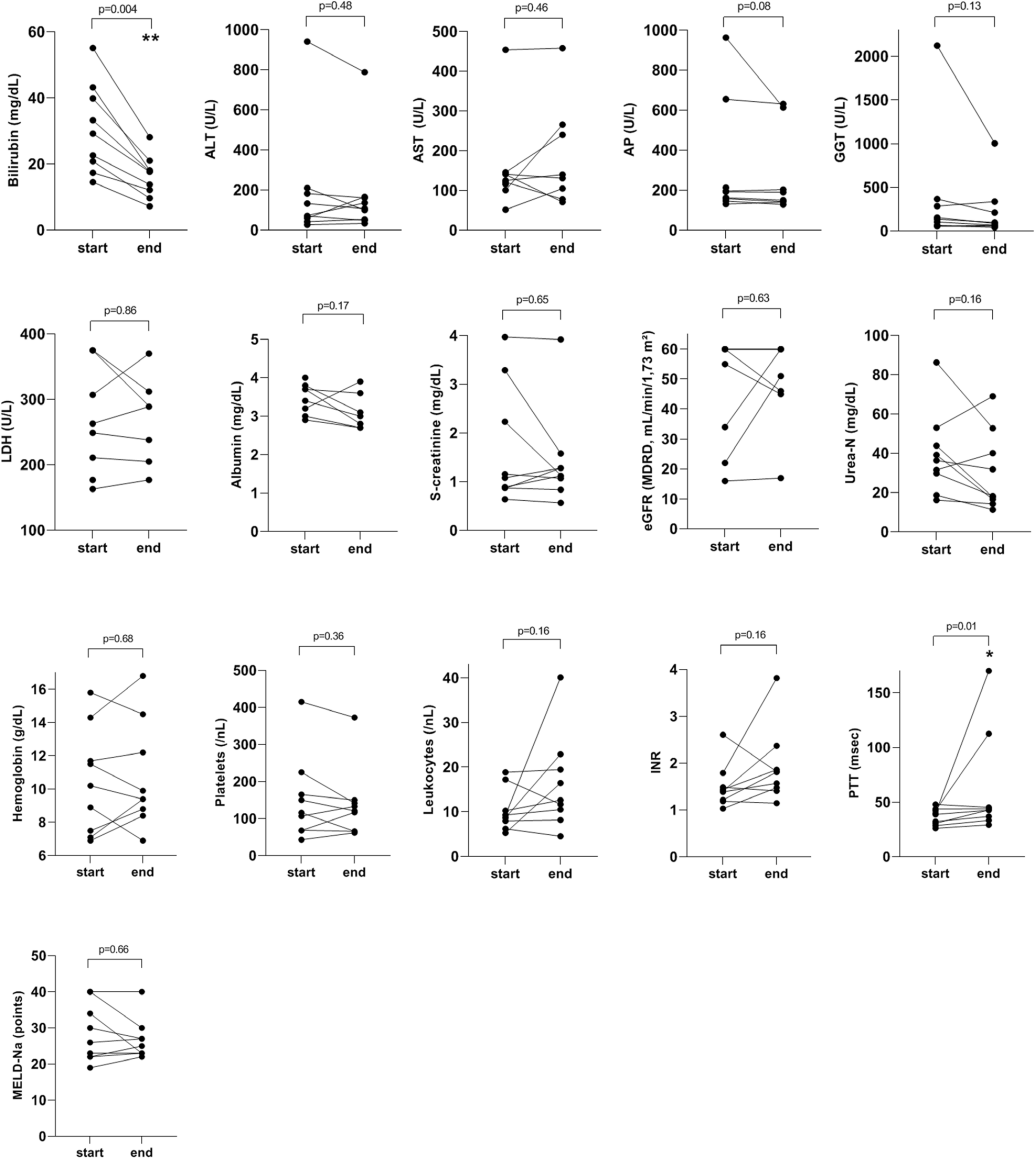
Changes in biochemical variables of 9 patients after the end of treatment with PAP using BR‐350. ALT, alanine transaminase; AP, alkaline phosphatase; AST, aspartate transaminase; INR, international normalized ratio; eGFR, estimated glomerular filtration rate; GGT, gamma‐glutamyltransferase; L, liter; LDH, lactate dehydrogenase; MDRD, modification of diet in renal disease; MELD‐Na, model for end‐stage liver disease‐ sodium; PAP, plasma adsorption perfusion; PTT, partial thromboplastin time; s‐creatinine, serum creatinine; U, unit; urea‐N, urea nitrogen.

**FIGURE 3:**
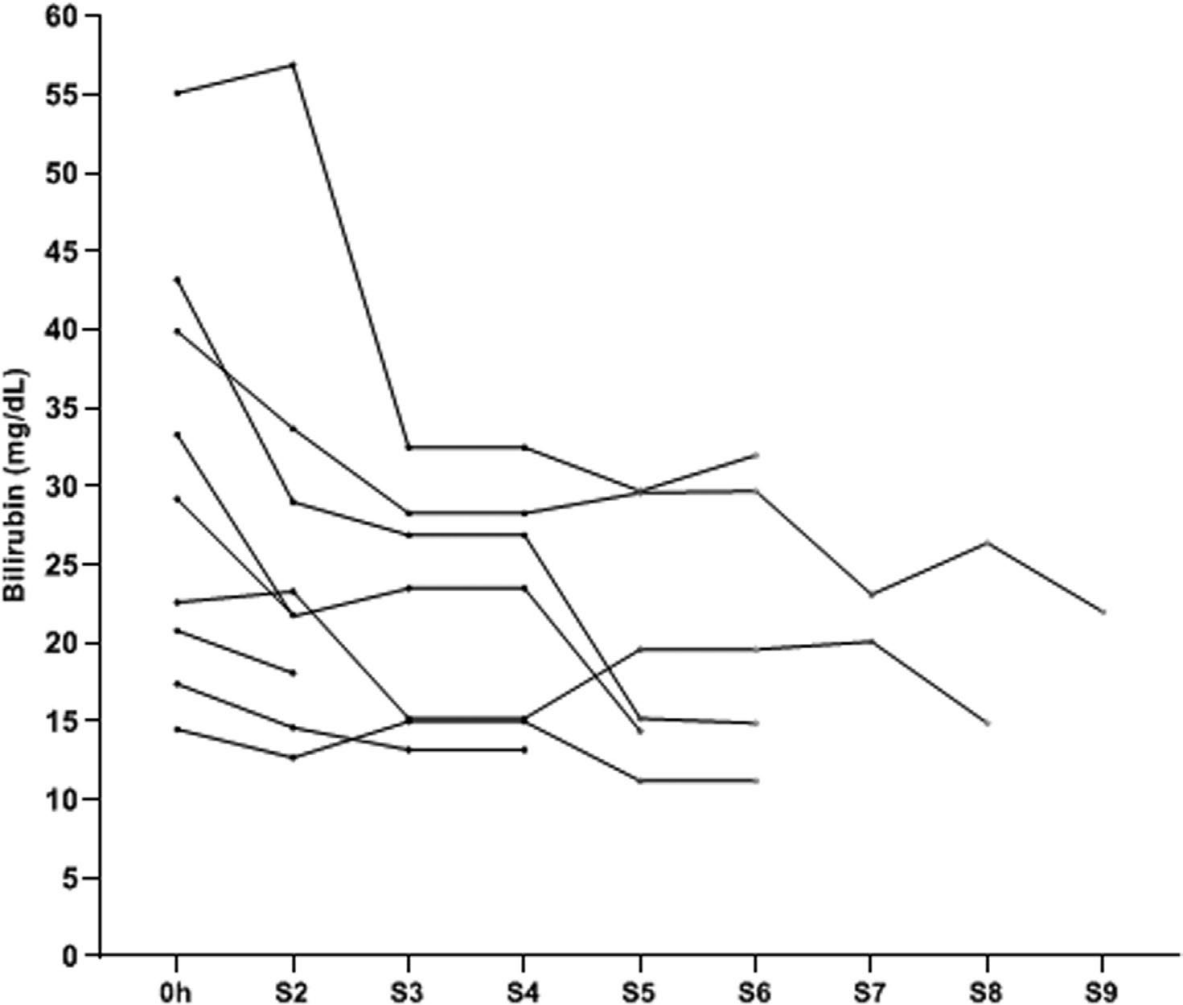
Individual courses of total serum bilirubin concentrations among 9 patients during the liver support with PAP using BR‐350. S, session.

Figure 1 illustrates the absolute change of bilirubin and other relevant laboratory values obtained within the first 24 h after the first session of PAP with BR‐350 among 9 patients. We observed a reduction of bilirubin levels already after the first session of PAP using BR‐350, whereas the other biochemical parameters did not significantly change (Figure 1). The relative reduction of bilirubin after the first session of PAP with BR‐350 was 16%. Figure 2 shows absolute changes of laboratory values in relation to the baseline values after completion of the liver support with PAP using BR‐350. Again, we found a significant decrease of bilirubin values after the end of liver support with PAP with BR‐350 (Figure 2). No paraclinical improvement of the liver function occurred under the treatment with PAP using BR‐350 (Figure 2). We documented a median relative reduction of bilirubin of 47% after the completion of PAP with BR‐350 (Table 2). The individual course of bilirubin concentrations during the extracorporeal therapy with PAP using BR‐350 was demonstrated for 9 patients in Figure 3.

**TABLE 2:** Relevant outcome parameters and complications in the cohort of 9 patients treated exclusively with PAP using BR‐350 between April 2016 and November 2023.

Variable	PAP with BR‐350
	*n* = 9
Relative reduction of bilirubin (%)	47.4 (39.2 to 51.9)
Relative reduction of MELD‐Na (%)	0 (−9.1 to 17.5)
Death within 30 days post treatment, *n* (%)	3 (33)
Transfer to ICU, *n* (%)	2 (22)
Liver transplant post treatment, *n* (%)	2 (22)
Bleeding under treatment, *n* (%)	1 (11)
Infection under treatment, *n* (%)	2 (22)

*Note:* Values are presented as medians with interquartile range.

Abbreviations: ICU, intensive care unit; MELD‐Na, model for end‐stage liver disease‐ sodium; PAP, plasma adsorption perfusion.

A bleeding event occurred in one patient under PAP with BR‐350 while two patients experienced an infection at the end of extracorporeal treatment (Table 2). Three patients died within the first 30 days after the completion of PAP with BR‐350 and two patients required transfer to the intensive care unit after the end of extracorporeal liver support (Table 2).

### 3.2 Liver Support Using PAP With BR‐350 Versus OPAL or SMT

In a next step, we aimed a comparison of PAP with BR‐350 with another liver support strategy, the OPAL, and with application of SMT including hemodialysis (Tables 3, 4, 5). The cohort of cirrhotic patients with diverse liver dysfunctions in which OPAL was applied consisted of 24 patients (Table 3). Considering baseline characteristics of the OPAL cohort and the above mentioned cohort of 9 patients, who received PAP with BR‐350, we saw that serum creatinine values tended to be higher and kidney failure with reduction of estimated GFR (eGFR) under 60 mL/min/1.73 m^2^ was more common among patients treated with OPAL than among patients treated with PAP with BR‐350 (Table 3). Other clinical and laboratory variables did not differ between the two groups (Table 3). We analyzed the relative change of relevant laboratory variables under treatment with PAP with BR‐350 and OPAL (Table 4). We found a more pronounced reduction of lactate dehydrogenase (LDH), hemoglobin, and leukocytes after liver support therapy with OPAL compared with PAP with BR‐350. The other studied variables, in particular parameters reflecting liver dysfunction, seemed to be comparable (Table 4). The relative bilirubin decrease was similar after the usage of both extracorporeal liver support options (Table 4). The 30‐day mortality rate, the number of patients requiring transfer to the intensive care unit, and the rate of the main complications such as bleeding and infections were also comparable between both liver support strategies (Table 5).

**TABLE 3:** Comparison of baseline characteristics between 9 patients treated with PAP using BR‐350 and 24 patients treated with OPAL and 24 patients who underwent SMT inclusive hemodialysis.

Variable	PAP with BR‐350	OPAL	RR (CI)	*p*	PAP with BR‐350	SMT	RR (CI)	*p*
	*n* = 9	*n* = 24			*n* = 9	*n* = 24		
Women, *n* (%)	5 (56)	6 (25)	1.86 (0.8–3.8)	0.12	5 (56)	11 (46)	1.21 (0.5–2.4)	0.62
Age (years)	54 (46–61)	51 (40–61)		0.52	54 (46–61)	50 (50–69)		0.12
Number of session	6 (4–7)	7 (3–9)		0.45	6 (4–7)	8 (3–11)		0.26
MELD‐Na score (points)	26 (22–37)	32 (28–37)		0.19	26 (22–37)	33 (28–37)		0.14
Hepatorenal syndrome, *n* (%)	3 (33)	14 (58)	0.57 (0.2–1.3)	0.20	3 (33)	24 (100)	0.33 (0.2–0.6)	**0.0001**
GFR ≥ 60 mL/min/1.73 m^2^ (MDRD), *n* (%)	5 (56)	6 (25)	2.22 (0.9–5.3)	0.10	5 (56)	0 (0)	infinity (2.0‐infinity)	**0.0001**
Hepatic encephalopathy, *n* (%)	3 (33)	9 (38)	0.89 (0.3–2.2)	0.82	3 (33)	12 (50)	0.68 (0.2–1.6)	0.39
Dialysis before therapy initiation, *n* (%)	0 (0)	5 (21)	0.0 (0.0–1.6)	0.14	0 (0)	6 (25)	0.0 (0.0–1.4)	0.10
Administration to the ICU before therapy initiation, *n* (%)	2 (22)	10 (42)	0.53 (0.2–1.6)	0.30	2 (22)	12 (50)	0.44 (0.1–1.3)	0.15
Another liver support device before therapy initiation, *n* (%)	0 (0)	2 (8)	0.0 (0.0–4.5)	0.37				
Etiology of liver dysfunction								
ACLF, *n* (%)	6 (67)	22 (92)	0.73 (0.4–1.1)	0.07	6 (67)	24 (100)	0.67 (0.4–0.9)	**0.03**
Decompensation of liver cirrhosis, *n* (%)	3 (33)	2 (8)	4.33 (0.95–19.0)	0.06	3 (33)	0 (0)	infinity (2.0‐infinity)	**0.003**
Baseline laboratory values								
Bilirubin (mg/dL)	29.2 (19.1–41.6)	28.7 (22.9–34.2)		0.83	29.2 (19.1–41.6)	7.2 (2.0–20.2)		**0.0008**
ALT (U/L)	74 (51–197)	63 (35–141)		0.39	74 (51–197)	42 (24–165)		0.17
AST (U/L)	125 (109–144)	169 (115–228)		0.11	125 (109–144)	77 (26–197)		0.22
AP (U/L)	193 (152–435)	185 (145–241)		0.77	193 (152–435)	127 (100–215)		**0.04**
GGT (U/L)	135 (60–325)	225 (107–496)		0.23	135 (60–325)	59 (123–203)		0.74
LDH (U/L)	256 (186–358)	272 (221–402)		0.47	256 (186–358)	283 (212–437)		0.43
INR	1.41 (1.21–1.64)	1.63 (1.38–2.19)		0.18	1.41 (1.21–1.64)	1.52 (1.32–1.98)		0.28
PTT (msec)	38.8 (29.6–43.0)	38.8 (29.9–48.4)		0.51	38.8 (29.6–43.0)	34.0 (43.0–53.0)		0.16
Albumin (mg/dL)	3.6 (3.1–3.8)	3.2 (2.8–3.6)		0.20	3.6 (3.1–3.8)	2.5 (3.1–3.3)		**0.03**
Serum creatinine (mg/dL)	1.08 (0.87–2.76)	2.04 (1.41–3.41)		0.08	1.08 (0.87–2.76)	4.99 (3.43–6.29)		**0.0001**
eGFR (MDRD, mL/min/1.73 m^2^)	60 (28–60)	35 (19–56)		0.11	60 (28–60)	12 (8–17)		**0.0001**
Urea‐N (mg/dL)	34.4 (25.3–48.5)	46.5 (24.5–82.9)		0.41	34.4 (25.3–48.5)	93.0 (60.4–142.5)		**0.0007**
Hemoglobin (g/dL)	10.2 (7.3–13.0)	9.3 (8.1–11.6)		0.88	10.2 (7.3–13.0)	8.4 (7.5–10.4)		0.40
Platelets (/nL)	116 (69–195)	137 (69–257)		0.77	116 (69–195)	97 (52–141)		0.22
Leukocytes (/nL)	9.3 (7.1–13.8)	12.1 (8.6–17.9)		0.19	9.3 (7.1–13.8)	12.3 (8.9–14.5)		0.21

*Note:* Values are presented as medians with interquartile range. Bold values represent statistical significant differences.

Abbreviations: ACLF, acute‐on‐chronic liver failure; ALT, alanine transaminase; AP, alkaline phosphatase; AST, aspartate transaminase; CI, confidence interval; eGFR, estimated glomerular filtration rate; GGT, gamma‐glutamyltransferase; INR, international normalized ratio; L, liter; LDH, lactate dehydrogenase; MDRD, modification of diet in renal disease; MELD‐Na, model for end‐stage liver disease‐ sodium; OPAL, open albumin dialysis; PAP, plasma adsorption perfusion; PTT, partial thromboplastin time; RR, relative risk; SMT, standard medical treatment; U, unit; urea‐N, urea nitrogen.

**TABLE 4:** Relative reduction of relevant laboratory values among 9 patients treated with PAP using BR‐350 versus 24 patients treated with OPAL and 24 patients who underwent SMT inclusive hemodialysis.

Relative reduction	PAP with BR‐350	OPAL	*p*	PAP with BR‐350	SMT	*p*
	*n* = 9	*n* = 24		*n* = 9	*n* = 24	
Bilirubin	47.4 (39 to 52)	40.0 (16 to 56)	0.29	47.4 (39 to 52)	−29.6 (−61 to 10)	**0.0001**
ALT	12.2 (−56 to 25)	14.3 (−14 to 46)	0.37	12.2 (−56 to 25)	10.0 (−35 to 38)	0.70
AST	−6.4 (−93 to 28)	22.9 (−25 to 46)	0.14	−6.4 (−93 to 28)	−6.0 (−59 to 26)	0.61
AP	6.0 (−3 to 21)	23.5 (−2 to 39)	0.13	6.0 (−3 to 21)	1.0 (−41 to 25)	0.68
GGT	34.3 (−10 to 43)	43.3 (18 to 64)	0.17	34.3 (−10 to 43)	10.3 (−41 to 46)	0.41
LDH	2.8 (−10 to 17)	22.5 (2 to 37)	**0.03**	2.8 (−10 to 17)	2.1 (−19 to 17)	0.81
INR	−24.6 (−56 to 12)	−13.0 (−27 to 7)	0.78	−24.6 (−56 to 12)	−5.6 (−23 to 12)	0.39
PTT	−14.2 (−103 to 5)	−9.0 (−41 to 1)	0.48	−14.2 (−103 to 5)	−12.7 (−39 to 15)	0.47
Albumin	10.0 (3 to 18)	1.5 (−9 to 15)	0.37	10.0 (3 to 18)	−4.6 (−25 to 13)	0.27
Serum creatinine	7.8 (−9 to 50)	19.8 (−2 to 42)	0.71	7.8 (−9 to 50)	16.7 (−18 to 36)	0.99
eGFR (MDRD)	0 (−41 to 9)	16.2 (−55 to 5)	0.56	0 (−41 to 9)	−28.6 (−68 to 14)	0.45
Urea‐N	38.6 (−8 to 49)	12.9 (−7 to 54)	0.71	38.6 (−8 to 49)	13.9 (−16 to 53)	0.69
Hemoglobin	−4.3 (−20 to 11)	23.7 (8 to 35)	**0.002**	−4.3 (−20 to 11)	13.4 (6 to 20)	**0.02**
Platelets	9.1 (−34 to 28)	15.9 (–23 to 51)	0.27	9.1 (−34 to 28)	26.7 (−21 to 51)	0.21
Leukocytes	−13.4 (−189 to 12)	19.4 (−9 to 31)	**0.04**	−13.4 (−189 to 12)	−14.4 (−23 to 14)	0.41
MELD‐Na	0 (−9.1 to 17.5)	10.3 (0 to 16)	0.22	0 (−9.1 to 17.5)	−2.7 (−11 to 5)	0.48

*Note:* Values are presented as medians with interquartile range. Bold values represent statistical significant differences.

Abbreviations: ALT, alanine transaminase; AP, alkaline phosphatase; AST, aspartate transaminase; eGFR, estimated glomerular filtration rate; GGT, gamma‐glutamyltransferase; INR, international normalized ratio; L, liter; LDH, lactate dehydrogenase; MDRD, modification of diet in renal disease; MELD‐Na, model for end‐stage liver disease‐ sodium; OPAL, open albumin dialysis; PAP, plasma adsorption perfusion; PTT, partial thromboplastin time; SMT, standard medical treatment; U, unit; urea‐N, urea nitrogen.

**TABLE 5:** Relevant outcome parameters and complications among 9 patients treated with PAP using BR‐350 versus 24 patients treated with OPAL and 24 patients who underwent SMT inclusive hemodialysis.

Variable	PAP with BR‐350 *n* = 9	OPAL *n* = 24	RR (CI)	*p*	PAP with BR‐350 *n* = 9	SMT *n* = 24	RR (CI)	*p*
Death within 30 days post treatment, *n* (%)	3 (33)	9 (38)	0.80 (0.3–2.1)	0.67	3 (33)	14 (58)	0.62 (0.2–1.4)	0.29
Transfer to ICU, *n* (%)	2 (22)	4 (17)	1.33 (0.3–5.1)	0.71	2 (22)	5 (21)	1.07 (0.3–3.8)	0.93
Liver transplant post treatment, *n* (%)	2 (22)	1 (4)	5.33 (0.7–37.5)	0.11	2 (22)	0 (0)	infinity (1.5‐infinity)	**0.02**
Bleeding under treatment, *n* (%)	1 (11)	5 (21)	0.53 (0.1–2.7)	0.52	1 (11)	6 (25)	0.44 (0.1–2.2)	0.38
Infection under treatment, *n* (%)	2 (22)	2 (8)	2.67 (0.5–13.2)	0.28	2 (22)	3 (13)	1.78 (0.4–7.5)	0.49

*Note:* Values are presented as medians with interquartile range. Bold values represent statistical significant differences.

Abbreviations: CI, confidence interval; ICU, intensive care unit; OPAL, open albumin dialysis; PAP, plasma adsorption perfusion; RR, relative risk; SMT, standard medical treatment.

In contrast to the PAP cohort, the historical cohort of a total of 24 cirrhotic patients who were exposed to SMT including hemodialysis experienced significantly more frequently a hepatorenal syndrome and an eGFR reduction under 60 mL/min/1.73 m^2^ (Table 3). Furthermore, patients who received SMT had significantly higher serum creatinine and urea‐nitrogen and lower eGFR, albumin and bilirubin values obtained at baseline than those patients who were treated with PAP with BR‐350 (Table 3). As expected, application of PAP with BR‐350 as liver support option led to a more efficient relative reduction of bilirubin values than SMT (Table 4). Further comparison of PAP using BR‐350 with SMT in terms of reduction of other relevant laboratory values revealed no statistically significant differences between the groups except for the higher decline of hemoglobin in the SMT group than in the PAP group (Table 4). There were no relevant differences in 30‐day survival rates and in the number of complication events between PAP with BR250 and SMT (Table 5).

### 3.3 Comparison Between PAP With BR‐350 and OPAL in a Cross‐Over Design

**FIGURE 4:**
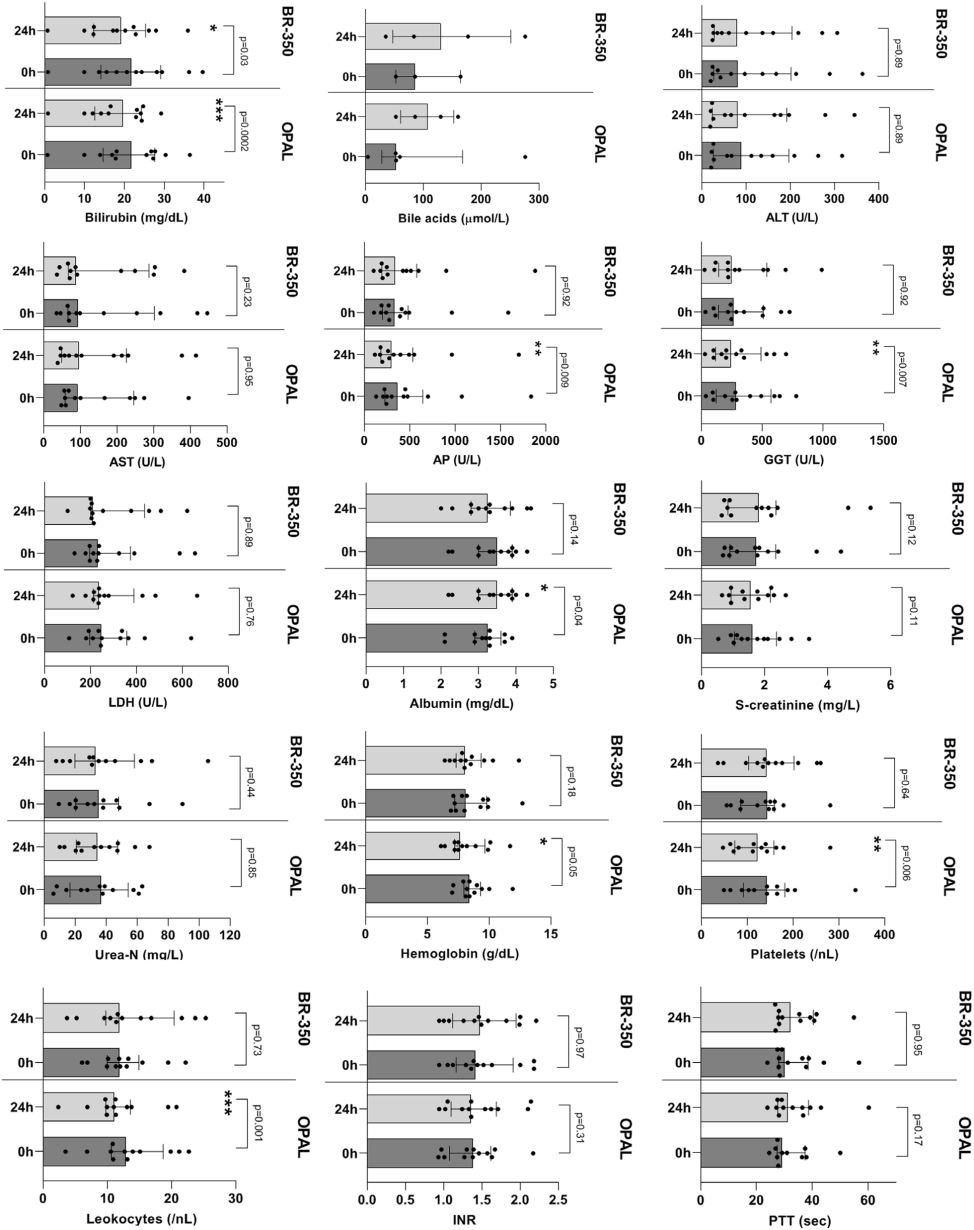
Changes in laboratory values of 12 patients after the single liver support session with OPAL and PAP using BR‐350 in a crossover design. ALT, alanine transaminase; AP, alkaline phosphatase; AST, aspartate transaminase; INR, international normalized ratio; eGFR, estimated glomerular filtration rate; GGT, gamma‐glutamyltransferase; L, liter; LDH, lactate dehydrogenase; MDRD, modification of diet in renal disease; MELD‐Na, model for end‐stage liver disease‐ sodium; OPAL, open albumin dialysis; PAP, plasma adsorption perfusion; PTT, partial thromboplastin time; s‐creatinine, serum creatinine; U, unit; urea‐N, urea nitrogen.

**FIGURE 5:**
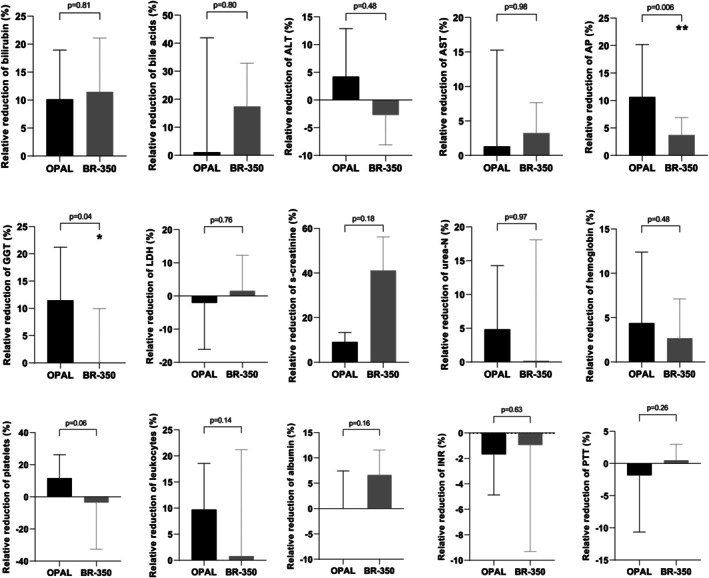
Comparison of relative changes in laboratory values after the single session of OPAL versus PAP using BR‐350 among 12 patients. ALT, alanine transaminase; AP, alkaline phosphatase; AST, aspartate transaminase; eGFR, estimated glomerular filtration rate; GGT, gamma‐glutamyltransferase; INR, international normalized ratio; L, liter; LDH, lactate dehydrogenase; MDRD, modification of diet in renal disease; MELD‐Na, model for end‐stage liver disease‐ sodium; OPAL, open albumin dialysis; PAP, plasma adsorption perfusion; PTT, partial thromboplastin time; s‐creatinine, serum creatinine; U, unit; urea‐N, urea nitrogen.

In addition, we performed a comparison between PAP with BR‐350 and OPAL in a cross‐over manner using another cohort containing a total of 12 cirrhotic patients having ACLF or therapy refractory pruritus needing extracorporeal liver support (Table 6). 5 (42%) of 12 patients had acute kidney injury (AKI) (Table 6). The median MELD‐Na score of the entire cohort was 27 points (Table 6). Seven (58%) patients received one session of OPAL first and afterwards one PAP session with BR‐350; in the remaining 5 (42%) patients, extracorporeal liver support therapy was performed vice versa (Table 6). For our comparison, we considered a single session of OPAL and PAP with BR‐350. Bilirubin values and other laboratory values measured at the beginning of the single liver support session were comparable between the OPAL and PAP group (Figure 4). Under the single OPAL session, we detected a significant decline of absolute values of bilirubin, alkaline phosphatase (AP), gamma‐glutamyltransferase (GGT), hemoglobin, platelets, and leukocytes, and a slight increase of albumin compared to the baseline values (Figure 4). Regarding the relative reduction of bilirubin, we observed a mean bilirubin reduction of 10% in the OPAL group, whereas the PAP session using BR‐350 resulted in a relative bilirubin reduction of 11% (Figure 5). The single OPAL session led to a higher relative reduction of AP, GGT, and hemoglobin in comparison to the single session of PAP with BR‐350 (Figure 5).

**TABLE 6:** Baseline characteristics of 12 patients who underwent a single liver support session with OPAL and PAP using BR‐350 in a crossover manner between March 2022 and May 2024.

Variable	All patients
	*n* = 12
Age (years), median (range)	51 (42–59)
Females, *n* (%)	7 (58)
OPAL as first treatment, *n* (%)	7 (58)
MELD‐Na score (points), median (range)	27 (23–32)
AKI, *n* (%)	5 (42)
Etiology of liver dysfunction	
ACLF, *n* (%)	10 (83)
Therapy refractory pruritus, *n* (%)	2 (17)

*Note:* Values are presented as medians with interquartile range.

Abbreviations: ACLF, acute‐on‐chronic liver failure; AKI, acute kidney injury; MELD‐Na, model for end‐stage liver disease‐ sodium; OPAL, open albumin dialysis; PAP, plasma adsorption perfusion.

**FIGURE 6:**
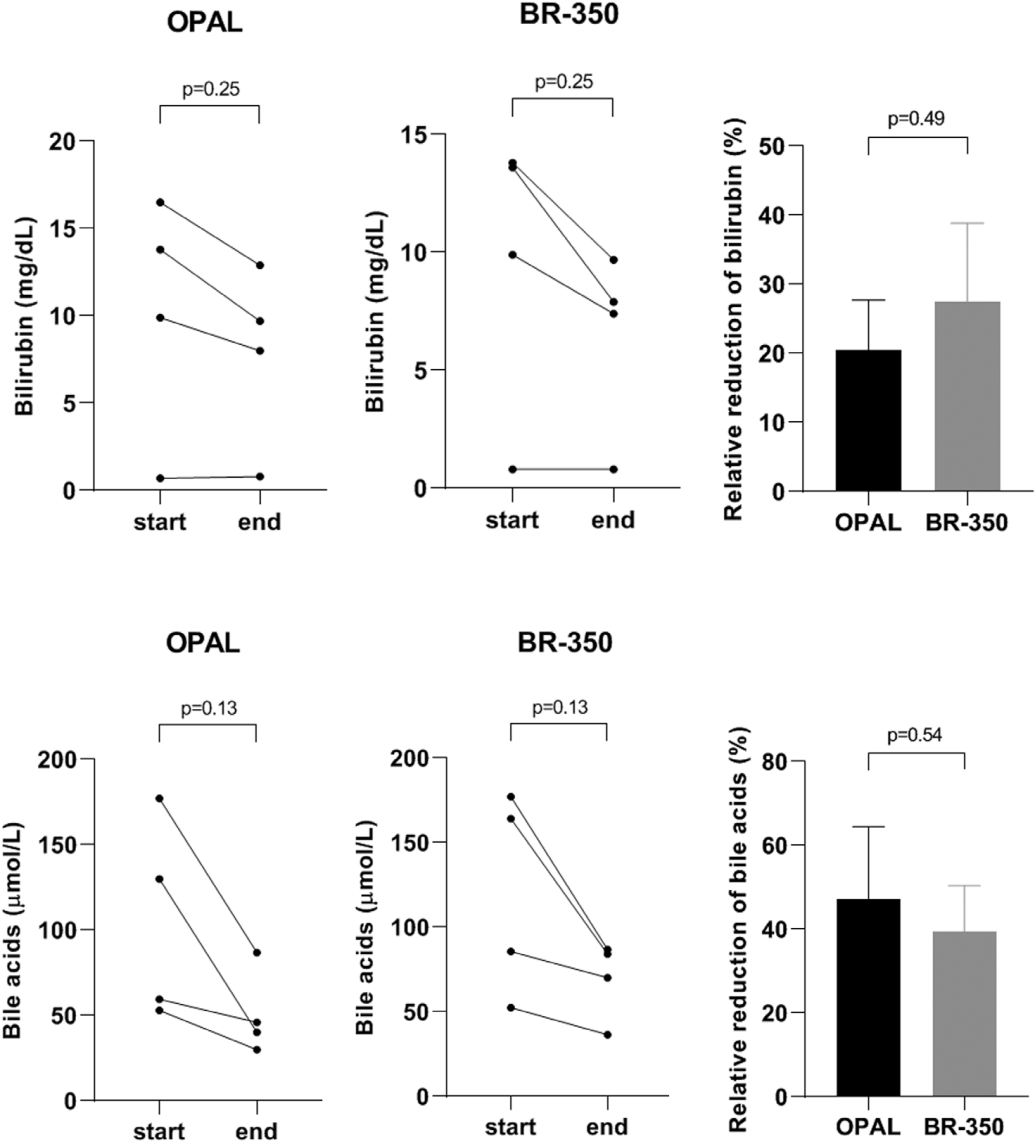
Absolute and relative changes in concentrations of bilirubin and bile acids measured at initiation and immediately at the end of the single session of OPAL versus PAP using BR‐350 among four patients. OPAL, open albumin dialysis; PAP, plasma adsorption perfusion.

In four of 16 patients, concentrations of bilirubin and bile acids were determined at the therapy start and immediately at the end of the single therapy session with OPAL and PAP (Figure 6). Both liver support devices were associated with a decline of the amount of bile acids (Figure 6). However, the extent of relative reduction of bile acid concentrations did not differ between the two liver support methods (Figure 6).

**FIGURE 7:**
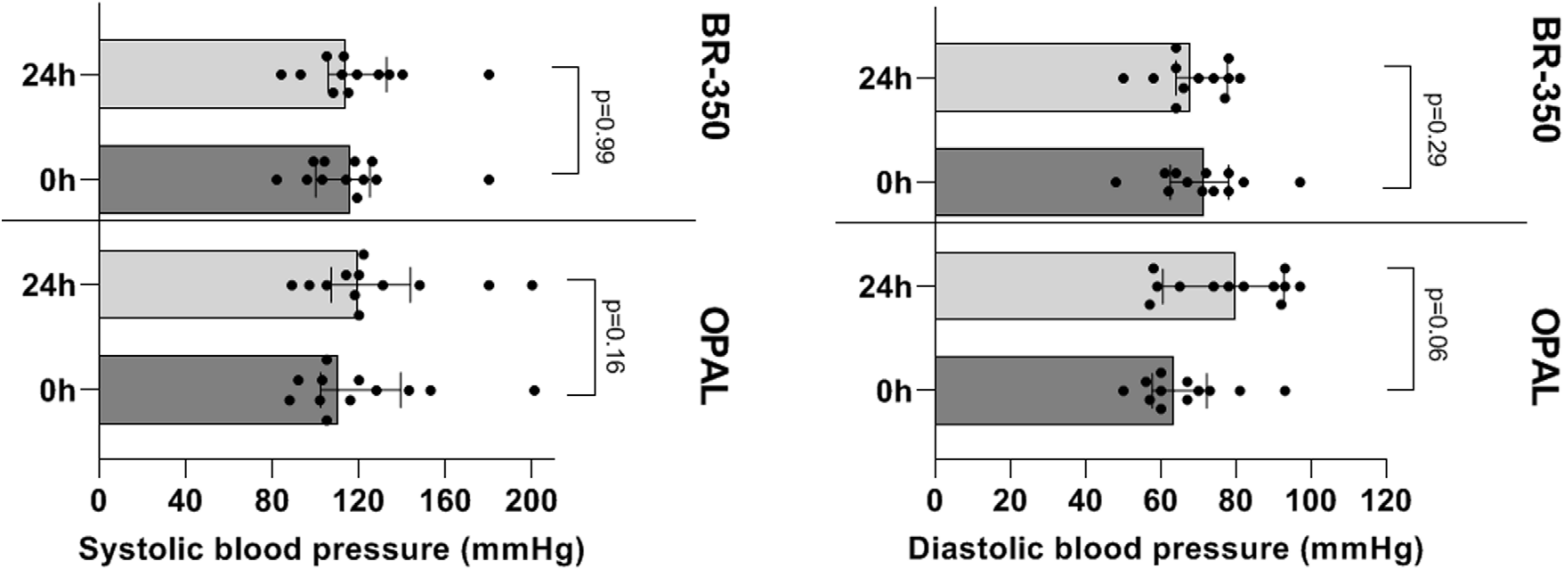
Changes in systolic and diastolic blood pressure of 12 patients during the single liver support session with OPAL and PAP using BR‐350 in a crossover design. OPAL, open albumin dialysis; PAP, plasma adsorption perfusion.

As presented in Figure 7, the single session of OPAL and PAP with BR‐350 did not significantly change the systolic and diastolic blood pressure (Figure 7).

## 4 Discussion

In the present work, we reported our previous experience with PAP using BR‐350 as a simple extracorporeal liver support method that does not require additional blood products in a cohort of 9 cirrhotic patients with acute impairment of hepatic function. The retrospective analysis revealed an efficient bilirubin removal with relative bilirubin reduction of 16% after the first single session of PAP using BR‐350. After the completion of the liver support therapy with PAP using BR‐350 consisting of an average six sessions we detected a relative decrease of bilirubin of 47%. The further retrospective comparison between PAP using BR‐350 and OPAL as an alternative liver support method based on albumin dialysis and performed among 24 cirrhotic patients showed comparable relative bilirubin reduction rates and comparable relative reduction of the MELD‐Na score after application of both liver support methods. OPAL was associated with a stronger reduction of hemoglobin, LDH, and leukocytes at the end of liver support treatment than PAP with BR‐350. As expected, the usage of PAP with BR‐350 was superior to the application of SMT in elimination of bilirubin. But regarding paraclinical improvement of the liver function, PAP with BR‐350 did not provide significantly better results than SMT alone. In the second step, we conducted a retrospective comparison between OPAL and PAP using BR‐350 in a crossover design in a cohort of 12 cirrhotic patients only considering the single session. We observed similar clearance of bilirubin using both devices in the same patients after the single session. However, the decrease of the concentrations of hemoglobin, platelets, and leukocytes was significantly larger after the single session of OPAL than after the single session of PAP.

Our results on bilirubin elimination capacity of PAP using BR‐350 are in line with the few available previous reports [17, 18, 19, 20]. The largest case series including 23 critically ill patients with hyperbilirubinemia related to liver failure of different origin, incorporating liver failures after liver transplant and partial liver resection, was reported by Senf et al. [19]. They demonstrated a statistically significant mean reduction of total bilirubin from 31 to 24 mg/dL (delta 7 mg/dL) after the performance of a single treatment of PAP with BR‐350 [19]. In our study, the mean bilirubin concentrations also significantly decreased from 31 to 26 mg/dL (delta 5 mg/dL) after the single PAP session. The lower difference between the pre‐ and post‐treatment values after the first session of PAP observed in our study in comparison to Senf et al. might be partly explained by lower bilirubin concentrations at baseline in our study than in the study of Senf et al. providing a lower gradient for bilirubin removal [19]. On the other hand, Senf et al. collected blood samples for bilirubin measurement immediately after every PAP cycle [19]. In case of our cohort of 9 patients, bilirubin measurement was performed routinely within the first 24 h after the end of each PAP session and mobilization of bilirubin from extravascular compartments to the blood circulation causing an elevation of plasma bilirubin levels might have occurred. We observed that serum concentrations of total bilirubin decreased from 31 to 16 mg/dL (delta 15 mg/dL) after the completion of on average six PAP cycles. Likewise, Adani et al. stated a decline of serum bilirubin from 31 to 15 mg/dL (delta 16 mg/dL) after three PAP sessions using BR‐350 among four patients with early cholestatic graft dysfunction in the setting of orthotopic liver transplantation [17].

The relative bilirubin reduction of 24% was achieved in the study of Senf et al. after the single session of PAP, whereas in our work total serum bilirubin lowered by 16% within the first 24 h after the first single treatment session of PAP with BR‐350 among 9 patients [19]. Considering four patients from the second cohort containing 12 patients in whom bilirubin concentrations were measured before and immediately after the single PAP session, a higher mean relative reduction of bilirubin of 24% was yielded. PAP with BR‐350 led to a relative reduction of total serum bilirubin ranging between 17% and 35% after each session in a report of Ott et al. that investigated bilirubin adsorption ability among two patients with excessive hyperbilirubinemia of more than 55 mg/dL developed due to a complicated liver transplant [20].

The research group of Senf et al. determined the decrease of bile acids of 20% after the single PAP session, while absolute concentrations of bile acids were significantly lowered from 42 to 34 mg/dL in several available patients [19]. In agreement with results of Senf et al., we detected a mean relative reduction of bile acids of 37% after performance of the single PAP therapy in four patients deriving from the cohort of 12 patients receiving PAP with BR‐250 and OPAL in a crossover manner. Bile acids concentrations were obtained immediately at the end of the single PAP session in these four patients just as in the study protocol of Senf et al. [19]. Hence, the greater relative reduction rate among our patients might be attributed to the fact that the plasma volume of 6–8 L separated per PAP session in our study was higher than the plasma volume of 3–4 L turned over in the case series of Senf et al. [19]. In contrast to our observations, Alarabi et al. did not find significant differences in circulating patient's serum bilirubin and bile acid levels obtained before and after PAP with BR‐350 analyzing four patients with intractable pruritus secondary to intrahepatic cholestasis [18]. However, Alarabi and colleagues reported a rapid reduction of the pruritus symptoms and a significant reduction of bilirubin and bile acids when comparing samples collected pre‐ and post‐adsorber column at each treatment session [18]. The authors argued that serum levels of bilirubin and bile acids remained unchanged after PAP because of the rapid replenishment of the substances from the mesenchymal tissue [18].

All previous reports suggested PAP with BR‐350 adsorber as a safe method of extracorporeal liver support [15, 16, 17, 18, 19, 20]. No major bleedings that might be caused by anticoagulation of the extracorporeal PAP system were documented [15, 16, 17, 18, 19, 20]. In the present cohort of 9 patients, subdural hematoma was diagnosed in one patient after the completion of PAP with BR‐350. We cannot fully exclude that this diagnosis might be a consequence of deteriorated hepatic function after liver transplant in this study subject. Additionally, blood culture was positive for *Staphylococcus aureus* in two patients from the current cohort at the end of PAP therapy indicating catheter infection.

To our knowledge, this is the first study comparing PAP using BR‐350 with OPAL. Recently, the research group of Riva et al. compared different extracorporeal liver support techniques including MARS, CytoSorb, and PAP with BR‐350 in terms of removal of bilirubin and bile acids [21]. However, they provided a limited comparison between PAP and other liver support devices because PAP with BR‐350 was conducted only in one patient [21]. In general, bilirubin reduction ranging between 30% and 40% and relative reduction of bile acids varying between 43% and 72% were described for the best studied albumin dialysis modality, MARS [21, 22, 23]. We saw comparable relative reduction rates for bilirubin after completion of OPAL (40%) and PAP (47%) as liver support therapies while the mean number of therapy sessions was higher in the OPAL group compared to the PAP group (7 vs. 5). A single session of PAP with BR‐350 resulted in a similar relative reduction of bilirubin as a single session of OPAL in the crossover comparison independently if the blood samples were collected within the first 24 h after the end of extracorporeal liver support session or immediately after the completion of the single liver support session. The relative reduction of bile acids also did not differ between the two liver support approaches. Opposed to the PAP cohort, the OPAL cohort of 24 patients contained a high proportion of patients with impaired renal function because the usage of OPAL with integrated option of hemodialysis was preferred in our center for the subgroup of patients with concomitant renal failure. PAP with BR‐350 was reserved for patients with hepatic failure and lack of renal impairment. This circumstance might have biased the results concerning clearance of the representative hydrophilic substances such as serum creatinine and urea nitrogen and might have led to the underestimation of the effect of OPAL. The improvement of serum creatinine levels among some cirrhotic patients exclusively treated with PAP using BR‐350 might be secondary and not directly related to the PAP treatment rather than to the overall improvement of the hepatorenal syndrome due to the stabilization of the liver function. The crossover comparison of the single session of OPAL with single session of PAP revealed no significant differences in removal of serum creatinine. From a technical background, PAP with BR‐350 adsorber is not able to remove water‐soluble toxins supporting the idea of exclusive use of OPAL for the subgroup of patients with combination of hepatic and renal failure. Indeed, PAP might be followed by hemodialysis when renal failure appears in addition to the hepatic dysfunction and OPAL device is not immediately available. We noted that a pronounced decrease of hemoglobin and platelets number occurred under OPAL treatment in comparison to the PAP. Therefore, application of PAP using BR‐350 might be considered as more beneficial for patients with a high risk of bleeding, in particular due to the hepatic failure.

However, comparing mortality rates under the two liver support approaches makes less sense because of the small number of patients and high heterogeneity of the hepatic failure and implications for extracorporeal liver support in both study cohorts. Removal of bilirubin and bile acids did not represent causal treatment and should be considered as a supportive therapy, whereas the overall prognosis is mainly influenced by the underlying disease that caused liver failure. Both liver support approaches showed high effectiveness in elimination of protein‐bound toxins. In contrast to the OPAL, PAP with BR‐350 did not require the addition of albumin and a long setup time, which makes this approach cost‐effective and justifies the use of PAP with BR‐350 as an extracorporeal treatment possibility to improve supportive therapy for hepatic failure with hyperbilirubinemia that did not respond to standard treatment. Bilirubin adsorption using PAP is a simple technique with a relatively short treatment time of 2–4 h per session that might be favored for the treatment of critically ill patients at the intensive care unit.

All abovementioned previous studies shared their experiences with PAP using BR‐350 without offering a comparison with standard medical treatment [15, 16, 17, 18, 19, 20]. Here, we attempted a comparison between the PAP cohort of 9 cirrhotic patients and 24 cirrhotic patients who received SMT combined with dialysis due to the hepatorenal syndrome. In opposite to the PAP group, bilirubin levels were relatively low with a median of 7 mg/dL in the SMT group and all patients belonging to the SMT group had acute kidney injury requiring hemodialysis, and as a consequence, they displayed higher disease severity. These visible differences might have impacted the data on the technical effectiveness, in particular on removal of bilirubin. Baseline bilirubin levels were significantly lower among patients who received SMT compared to those patients who were treated with PAP or OPAL. In this case, the selection bias plays a role because patients having hyperbilirubinemia were given preference for extracorporeal liver support therapy instead of SMT in order to modify bilirubin levels.

We are aware of the several limitations of our study besides the small number of study patients who underwent the different liver support therapies. To our opinion, the main limitation represents the fact that we used laboratory data that were obtained retrospectively in clinical routines to evaluate the detoxification capability of PAP with BR‐350 and OPAL. In the majority of patients, the blood samples were collected within the 24 h after the end of the single liver support cycle. Unfortunately, analysis of post‐treatment measurements performed directly after the liver support cycle was possible only in some patients. The optimal way would be determination of the adsorption rate of bilirubin and bile acids when taking blood before and after the adsorber at the initiation and at the end of the liver support session. Nevertheless, our analysis is based on a homogenous study population including only cirrhotic patients with ACLF or decompensation of the preexisting liver cirrhosis that represents a strength of our study. Further prospective studies with accordingly optimized study settings and a higher number of participants are required to address the kinetics of removal of bilirubin and bile acids under PAP with BR‐350 in comparison to albumin dialysis such as OPAL.

We consider it important to report our experience on the technical effectiveness of PAP with BR‐350 as an extracorporeal liver support for cirrhotic patients with hepatic failure and hyperbilirubinemia. With regard to the clearance of bilirubin and bile acids, the PAP using BR‐350 was not inferior to the OPAL and could be taken into account as an alternative to albumin dialysis in patients who present with normal renal function. PAP using BR‐350 may not require close monitoring of platelets and hemoglobin. Finally, the current study indicates that the PAP device should be reserved for cirrhotic patients with hepatic dysfunction without occurrence of renal impairment because this method does not allow an effective removal of hydrophilic toxins.

## Author Contributions

Concept/design: Justa Friebus‐Kardash, Andreas Kribben, Amos Zeller, Bartosz Tyczynski, and Hartmut H. Schmidt. Data analysis/interpretation: Justa Friebus‐Kardash, Amos Zeller, Alica Ochs, Amina Louzi, Jassin Rashidi‐Alavijeh, and Andreas Schütte. Drafting article: Justa Friebus‐Kardash and Amos Zeller. Critical revision of article: Andreas Kribben, Bartosz Tyczynski, Hartmut H. Schmidt, Jassin Rashidi‐Alavijeh, and Andreas Schütte. Statistics: Justa Friebus‐Kardash, Amina Louzi, and Alica Ochs. Data collection: Justa Friebus‐Kardash, Amina Louzi, Alica Ochs, Amos Zeller, and Andreas Schütte. Funding secured by: Hartmut H. Schmidt and Andreas Kribben. Supervision: Justa Friebus‐Kardash. Project administration: Justa Friebus‐Kardash, Amos Zeller, Bartosz Tyczynski, Andreas Schütte, and Jassin Rashidi‐Alavijeh.

## Funding

The authors have nothing to report.

## Ethics Statement

The study was conducted according to the guidelines of the Declaration of Helsinki and was approved by the Ethics Committee of the University Hospital Essen (23‐11405‐BO).

## Consent

The authors have nothing to report.

## Conflicts of Interest

The authors declare no conflicts of interest.

## Data Availability

The data that support the findings of this study are available on request from the corresponding author. The data are not publicly available due to privacy or ethical restrictions.
